# Poster Session II A335 FLNA DEFICIENCY PRESENTING AS INTESTINAL PSEUDO-OBSTRUCTION IN INFANCY: A CASE REPORT AND LITERATURE REVIEW.

**DOI:** 10.1093/jcag/gwaf042.335

**Published:** 2026-02-13

**Authors:** W E Alrasheed, M Sherlock, E Ratcliffe, R Issenman, L VanHouwelingen, N Lepore

**Affiliations:** Pediatric Gastroenterology, McMaster University Faculty of Health Sciences, Hamilton, ON, Canada; Pediatric Gastroenterology, McMaster University Faculty of Health Sciences, Hamilton, ON, Canada; Pediatric Gastroenterology, McMaster University Faculty of Health Sciences, Hamilton, ON, Canada; Pediatric Gastroenterology, McMaster University Faculty of Health Sciences, Hamilton, ON, Canada; Pediatric Gastroenterology, McMaster University Faculty of Health Sciences, Hamilton, ON, Canada; Pediatric Gastroenterology, McMaster University Faculty of Health Sciences, Hamilton, ON, Canada

## Abstract

**Background:**

Filamin A (FLNA) is a cytoplasmic actin cross-linking protein that plays a crucial role in maintaining cytoskeletal integrity and regulating cell motility and migration. Pathogenic variants in the FLNA gene are associated with a wide phenotypic spectrum including neurological, cardiovascular, and gastrointestinal manifestations. Gastrointestinal dysmotility, including chronic intestinal pseudo-obstruction (CIPO), represents one of the less common but significant features of *FLNA*-related disorders. Recognizing these presentations is essential for early diagnosis and tailored management.

**Aims:**

To report a rare case of FLNA deficiency presenting as intestinal pseudo-obstruction in infancy and to review the related literature.

**Methods:**

We conducted a case report of a six-month-old male presenting with features of intestinal obstruction. A literature review was performed to contextualize the findings and explore genotype-phenotype correlations.

**Results:**

The infant presented with poor oral intake, weight loss, progressive abdominal distention, recurrent vomiting, and profound failure to thrive. Examination revealed malnutrition and abdominal distention with decreased bowel sounds. Imaging demonstrated small bowel dilatation and malrotation without volvulus. Exploratory laparotomy found no mechanical obstruction, but the bowel was significantly dilated throughout. A Ladd’s procedure with ileostomy was performed. Full thickness biopsies of the ileum showed normal smooth muscle morphology. Genetic testing identified a pathogenic hemizygous FLNA variant (c.18_19del; p.Arg7GlyfsTer98), confirming FLNA deficiency. Brain MRI revealed periventricular nodular heterotopia. The patient remains on combined parenteral and enteral nutrition. The *FLNA* c.18_19del (p.Arg7GlyfsTer98) variant in our patient has been previously reported in males with CIPO. This deletion disrupts the long FLNA isoform, predominantly expressed in intestinal smooth muscle, resulting in impaired contractility and myopathic intestinal dysmotility. Experimental models confirm the long isoform’s essential role in intestinal elongation and peristalsis. The gastrointestinal phenotype of FLNA deficiency varies widely, from constipation to severe CIPO with malrotation and feeding intolerance.

**Conclusions:**

This case highlights the variable presentation of FLNA deficiency and the importance of considering FLNA mutations in males with early onset intestinal pseudo-obstruction. Early diagnosis supports individualized management and genetic counseling, while future reports of similar cases can enhance understanding of this rare disorder.

**Funding Agencies:**

None

